# Psychological distress mediates the association between COVID-19-related discrimination and subsequent PTSD symptom severity in healthcare workers: a two-year follow-up study

**DOI:** 10.1186/s12889-024-19359-z

**Published:** 2024-07-09

**Authors:** Zui Narita, Ryo Okubo, Yohei Sasaki, Kazuyoshi Takeda, Masaki Takao, Hirofumi Komaki, Hideki Oi, Takeshi Miyama, Yoshiharu Kim

**Affiliations:** 1 Department of Behavioral Medicine, National Institute of Mental Health, National Center of Neurology and Psychiatry, 4-1-1 Ogawahigashicho, Kodaira, Tokyo 187-8553 Japan; 2 Clinical Research & Education Promotion Division, National Center of Neurology and Psychiatry Hospital, 4-2-2 Ogawahigashicho, Kodaira, Tokyo 187-8551 Japan; 3 National Center of Neurology and Psychiatry Hospital, 4-1-1 Ogawahigashicho, Kodaira, Tokyo 187-8551 Japan

**Keywords:** Post-Traumatic stress disorder, SARS-CoV-2, Social isolation, Social stigma, Psychological trauma

## Abstract

**Background:**

Past research has suggested a cross-sectional association between COVID-19-related discrimination and PTSD symptom severity. However, no cohort study has examined the longitudinal association that better supports causal interpretation. Also, even if such an association genuinely exists, the specific pathway remains unclear.

**Methods:**

We conducted a two-year follow-up study, obtaining data from healthcare workers in a hospital setting. We first evaluated how COVID-19-related discrimination in 2021 was associated with subsequent PTSD symptom severity in 2023. Thereafter, we conducted causal mediation analysis to examine how this association was mediated by psychological distress in 2022, accounting for exposure-mediator interaction. Missing data were handled using random forest imputation.

**Results:**

A total of 660 hospital staff were included. The fully adjusted model showed greater PTSD symptom severity in individuals who experienced any COVID-19-related discrimination compared with those without such experiences (*β*, 0.44; 95% CI, 0.04–0.90). Regarding each type of discrimination, perceived discrimination was associated with greater PTSD symptom severity (*β*, 0.52; 95% CI, 0.08–0.96), whereas verbal discrimination did not reach statistical significance. Psychological distress mediated 28.1%–38.8% of the observed associations.

**Conclusions:**

COVID-19-related discrimination is associated with subsequent PTSD symptom severity in healthcare workers. Psychological distress may serve as an important mediator, underscoring the potential need for interventions targeting this factor.

## Background

The COVID-19 pandemic has substantially influenced the physical and psychological well-being of populations globally [1]. Beyond the direct impact on physical health, there are also concerns regarding the mental health consequences of the pandemic [2]. In particular, healthcare workers treating COVID-19 patients experienced numerous challenges, including extreme workloads, shortages of drugs and protective gear, and the necessity of self-isolation [3]. These factors may have contributed to adverse mental health outcomes including PTSD [4]. A meta-analysis suggested a high prevalence of PTSD symptoms among healthcare workers during the pandemic [5].

Discrimination adversely affects mental health [6], and this might be especially relevant in healthcare workers given the high probability of experiencing discrimination [7–10]. COVID-19-related discrimination against healthcare workers was shown to be associated with poorer mental health [11–14]. Of these, one cross-sectional study suggested the association between COVID-19-related discrimination and greater PTSD symptom severity [14]. Nevertheless, two limitations merit attention in this area of research. First, no cohort study has examined the longitudinal association that better supports causal interpretation. Second, even if such an association genuinely exists, the specific pathway remains unclear. This is particularly pertinent because COVID-19-related discrimination per se may not fulfill the criterion A for PTSD defined by the DSM-5, i.e., exposure to actual or threatened death, serious injury, or sexual violence [15], and thus an indirect pathway towards PTSD symptom severity should be considered. Past research suggested that COVID-19-related discrimination was associated with psychological distress [13, 14]. Furthermore, psychological distress was associated with COVID-19-related traumatic stress [16]. Thus, it is conceivable that COVID-19-related discrimination may initially lead to increased psychological distress, which, in turn, could result in greater severity of traumatic stress symptoms.

To address the aforementioned limitations, we conducted a cohort study with a two-year follow-up, obtaining data from healthcare workers in a hospital setting. We first evaluated how COVID-19-related discrimination in 2021 was associated with subsequent PTSD symptom severity in 2023. Thereafter, we examined how this association was mediated by psychological distress in 2022. Data from three time points are necessary for such analysis to better support causal interpretation, evaluating the exposure, mediator, and outcome at different time points. While the association between COVID-19-related discrimination and PTSD symptom severity from the cross-sectional study [14] did not corroborate causal associations, reverse causation appeared unlikely given that PTSD symptoms would not cause COVID-19-related discrimination. Therefore, we hypothesized that COVID-19-related discrimination was associated with greater PTSD symptom severity. We also hypothesized that this association was partly mediated by psychological distress considering the potential pathway mentioned above.

## Methods

### Study population

**Fig.1:**
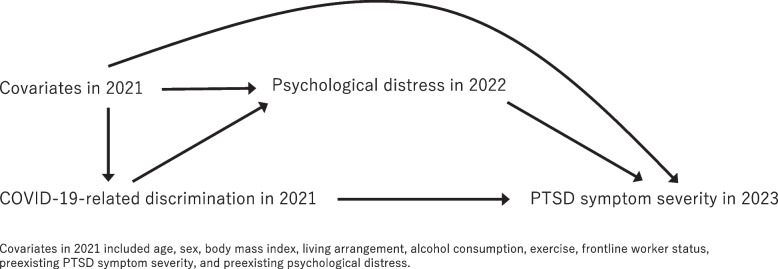
Directed acyclic graph for the mediation analysis

This cohort study with a two-year follow-up used data from staff at a national hospital in Japan. Hospital staff were invited to complete questionnaires on COVID-19-related discrimination (exposure) and covariates as baseline data in February 2021. Follow-up data were collected in January 2022 for psychological distress (mediator) and in September 2023 for PTSD symptom severity (outcome). The directed acyclic graph of the hypothesized associations is shown in Fig. 1. The inclusion criterion was that all individuals participated in the survey, and no exclusion criteria were implemented. The study was reviewed and approved by the Institutional Review Board of the National Center of Neurology and Psychiatry (A2020-121). All participants provided informed consent to participate in the study.

### COVID-19-related discrimination (exposure)

COVID-19-related discrimination was assessed by asking participants two questions with a yes/no answer option, previously used in other studies [13, 14, 17, 18]: (1) “Have you or your family ever experienced verbal discrimination related to COVID-19?” and (2) “Have you ever perceived discrimination related to COVID-19?”. Endorsing either of these experiences was labeled as any discrimination.

### PTSD symptom severity (outcome)

PTSD symptom severity in 2021 and 2023 was evaluated using the three-item Posttraumatic Diagnostic Scale (PDS) [19], a validated scale derived from the original PDS [20]. This scale assesses symptom severity over the past month, corresponding to criteria B1 (intrusive images), B2 (nightmares), and B5 (physical reactions when reminded of the trauma) [19]. Each item was self-reported using a four-point response scale from 0 (not at all or only one time) to 3 (five or more times a week/almost always), with possible scores from 0 to 9. Higher scores suggest greater PTSD symptom severity. Our data demonstrated good internal consistency (Cronbach’s alpha, 0.84).

### Covariates

We applied the modified disjunctive cause criterion to control for potential causes of COVID-19-related discrimination, PTSD symptom severity, or both, excluding instrumental variables and including covariates that served as proxies for unmeasured variables that are common causes of both the COVID-19-related discrimination and PTSD symptom severity [21]. We included the following covariates in 2021: age (continuous), sex (dichotomous, female or male), body mass index (continuous), living arrangement (dichotomous, living with someone or living alone) [22], alcohol consumption (dichotomous, < once a week or ≥ once a week) [23], exercise (dichotomous, < one hour/week or ≥ one hour/week) [24], frontline worker status (dichotomous, second-line or frontline) [8]. To address the possibility of reverse causation, we further included preexisting PTSD symptom severity in 2021 (continuous). Frontline worker status was ascertained by asking participants the following question with a yes/no answer option: “Have you ever engaged in COVID-19-related work?”. Participants were also asked to select the single occupation in which they spent the most time from the following answer options: (1) administrators, (2) physicians, (3) nurses, (4) medical staff other than office workers, (5) medical office workers, (6) other office workers, (7) information technology officers, (8) researchers, (9) janitors or security officers, and (10) other jobs. Those who answered “yes” to the first question and selected either (2), (3), or (4) in the second question were classified as frontline workers, while other individuals were considered as second-line workers.

### Psychological distress (mediator)

We evaluated psychological distress in 2021 and 2022 a priori based on the aforementioned theory. Psychological distress was measured using the Six-item Kessler Psychological Distress Scale (K6) [25]. Each item was self-reported on a five-point scale from 0 (no distress) to 4 (maximum distress), with total possible scores from 0 to 24. Higher scores suggest greater psychological distress. Our data showed good internal consistency (Cronbach’s α, 0.89).

### Statistical analysis

All analyses were conducted using R 4.3.2. We used generalized linear models to evaluate the association between COVID-19-related discrimination in 2021 and subsequent PTSD symptom severity in 2023. We evaluated three models, i.e., unadjusted, age, sex-adjusted, and fully adjusted models. The fully adjusted model included all of the aforementioned covariates. Random forest imputation was used [26] to address missing data for all relevant variables.

Next, we applied causal mediation analysis [27] to examine how the association between COVID-19-related discrimination in 2021 and PTSD symptom severity in 2023 was mediated by psychological distress in 2022. In the mediation analysis, psychological distress in 2021 was controlled for, in addition to the aforementioned covariates. We used the CMAverse package [28], accounting for an exposure-mediator interaction. This approach estimated the pure direct and total indirect effects, with the sum of these effects constituting the total effect. Bootstrap confidence intervals (CIs) for these effects were obtained using percentiles from 1000 samples. The proportion mediated was calculated as the total indirect effect divided by the total effect.

## Results

Table 1 summarizes the baseline characteristics of the study population in 2021, categorized by experiences of COVID-19-related discrimination. A total of 660 participants were included. The mean age (standard deviation) was 42.7 (10.9) years, and 446 (67.6%) of the participants were female. Data on COVID-19-related discrimination were missing for six individuals (0.9%). Individuals who experienced COVID-19-related discrimination were more likely to be younger, female, have a higher body mass index, live alone, consume alcohol more frequently, exercise regularly, and notably, work as frontline workers (39.6% vs. 19.5%) than those without such experiences. Moreover, the degree of PTSD symptoms and psychological distress was higher in individuals who experienced COVID-19-related discrimination. In both the 2022 and 2023 follow-ups, 192 participants completed the survey.

**Table 1:** Baseline characteristics of the study population in 2021 by COVID-19-related discrimination

		COVID-19-related discrimination		
	Overall (*N* = 660)	No (*N* = 606)	Yes (*N* = 48)	Missing (*N* = 6)
Age, mean (SD), y	42.7 (10.9)	42.9 (11.0)	39.6 (9.85)	43.3 (5.03)
Missing	4 (0.6)	1 (0.2)	0 (0)	3 (50.0)
Sex, no. (%)				
Male	210 (31.8)	195 (32.2)	13 (27.1)	2 (33.3)
Female	446 (67.6)	410 (67.7)	35 (72.9)	1 (16.7)
Missing	4 (0.6)	1 (0.2)	0 (0)	3 (50.0)
Body mass index, mean (SD)	22.2 (3.45)	22.1 (3.38)	23.3 (4.31)	22.4 (0.694)
Missing	13 (2.0)	7 (1.2)	3 (6.3)	3 (50.0)
Living arrangement, no. (%)				
Living with someone	496 (75.2)	462 (76.2)	33 (68.8)	1 (16.7)
Living alone	159 (24.1)	142 (23.4)	15 (31.3)	2 (33.3)
Missing	5 (0.8)	2 (0.3)	0 (0)	3 (50.0)
Alcohol consumption, no. (%)				
< once a week	372 (56.4)	350 (57.8)	21 (43.8)	1 (16.7)
≥ once a week	284 (43.0)	256 (42.2)	26 (54.2)	2 (33.3)
Missing	4 (0.6)	0 (0)	1 (2.1)	3 (50.0)
Exercise, no. (%)				
< one hour/week	425 (64.4)	395 (65.2)	28 (58.3)	2 (33.3)
≥ one hour/week	232 (35.2)	211 (34.8)	20 (41.7)	1 (16.7)
Missing	3 (0.5)	0 (0)	0 (0)	3 (50.0)
Frontline worker status, no. (%)				
Second-line worker	517 (78.3)	486 (80.2)	29 (60.4)	2 (33.3)
Frontline worker	138 (20.9)	118 (19.5)	19 (39.6)	1 (16.7)
Missing	5 (0.8)	2 (0.3)	0 (0)	3 (50.0)
PTSD symptoms, mean (SD)	0.850 (1.53)	0.795 (1.50)	1.52 (1.82)	1.33 (1.15)
Missing	26 (3.9)	21 (3.5)	2 (4.2)	3 (50.0)
Psychological distress, mean (SD)	4.75 (4.62)	4.55 (4.50)	6.98 (5.23)	8.67 (8.33)
Missing	6 (0.9)	3 (0.5)	0 (0)	3 (50.0)

*SD* Standard deviation

Table 2 summarizes the association between COVID-19-related discrimination in 2021 and subsequent PTSD symptom severity in 2023. The fully adjusted model showed greater PTSD symptom severity in individuals who experienced any COVID-19-related discrimination compared with those without such experiences (*β*, 0.44; 95% CI, 0.04 to 0.90). Findings were similar throughout the adjustments: unadjusted, age, sex-adjusted, and fully adjusted models. When examining each type of discrimination, perceived discrimination was associated with greater PTSD symptom severity (*β*, 0.52; 95% CI, 0.08 to 0.96), whereas verbal discrimination did not reach statistical significance.

**Table 2:** Association between COVID-19-related discrimination in 2021 and subsequent PTSD symptoms in 2023

	Unadjusted *β* [95% CI]	Age, sex-adjusted *β* [95% CI]	Fully adjusted *β* [95% CI]
Any discrimination			
No	0.00 (Reference)	0.00 (Reference)	0.00 (Reference)
Yes	0.47 [0.05, 0.90]	0.44 [0.01, 0.87]	0.47 [0.04, 0.90]
Verbal discrimination			
No	0.00 (Reference)	0.00 (Reference)	0.00 (Reference)
Yes	0.06 [-0.35, 0.47]	0.06 [-0.78, 0.89]	0.31 [-0.52, 1.13]
Perceived discrimination			
No	0.00 (Reference)	0.00 (Reference)	0.00 (Reference)
Yes	0.55 [0.10, 0.99]	0.51 [0.07, 0.96]	0.52 [0.08, 0.96]

CI, confidence interval

Missing data were handled using random forest imputation

All analyses were adjusted for the following covariates in 2021: age, sex, body mass index, living arrangement, alcohol consumption, exercise, frontline worker status, and preexisting PTSD symptom severity

Table 3 summarizes the mediating role of psychological distress in 2022 on the association between COVID-19-related discrimination in 2021 and subsequent PTSD symptom severity in 2023. A reasonable proportion was consistently mediated by psychological distress when evaluating any (33.8%), verbal (38.8%), and perceived (28.1%) discrimination.

**Table 3:** Mediating role of psychological distress in 2022 on the association between COVID-19-related discrimination in 2021 and subsequent PTSD symptoms in 2023

Exposure	Total effect *β* [95% bootstrap CI]	Pure direct effect *β* [95% bootstrap CI]	Total indirect effect *β* [95% bootstrap CI]	Proportion mediated, %
Any discrimination	0.28 [-0.09, 0.64]	0.18 [-0.15, 0.55]	0.09 [-0.03, 0.23]	33.8
Verbal discrimination	0.17 [-0.45, 1.04]	0.10 [-0.49, 0.95]	0.06 [-0.18, 0.36]	38.8
Perceived discrimination	0.31 [-0.07, 0.79]	0.22 [-0.16, 0.68]	0.09 [-0.04, 0.25]	28.1

CI, confidence interval

Missing data were handled using random forest imputation

All analyses were adjusted for the following covariates in 2021: age, sex, body mass index, living arrangement, alcohol consumption, exercise, frontline worker status, preexisting PTSD symptom severity, and preexisting psychological distress

The CIs for pure direct and total indirect effects were calculated using the percentiles from 1000 bootstrapped samples

Proportion mediated was calculated by total indirect effect/total effect

## Discussion

As hypothesized, COVID-19-related discrimination was associated with subsequent PTSD symptom severity in hospital staff. The findings were consistent throughout the models with different adjustments. While our findings align with the previous cross-sectional study suggesting the association between COVID-19-related discrimination and PTSD symptom severity [14], this study is the first to show a longitudinal association that better supports causal interpretations.

For PTSD symptom severity, we evaluated intrusive images, nightmares, and physical reactions when reminded of the trauma, corresponding to B1, B2, and B5 criteria, respectively. Note that we did not evaluate other symptoms, namely reliving of the trauma (B3) and being emotionally upset when reminded of the trauma (B4). While COVID-19-related discrimination was associated with subsequent PTSD symptom severity, the discrimination per se may not fulfill the criterion A for PTSD defined by DSM-5, i.e., exposure to actual or threatened death, serious injury, or sexual violence [15]. In this context, our study examined the indirect pathway from COVID-19-related discrimination to PTSD symptom severity and showed that 28.1%–38.8% of the association was indeed mediated by psychological distress. Thus, such discrimination may initially lead to increased psychological distress, which, in turn, may result in greater PTSD symptom severity. Our study adds evidence on the specific pathway between COVID-19-related discrimination and subsequent PTSD symptom severity. Furthermore, our findings may help guide future studies investigating the mechanism for other kinds of discrimination associated with PTSD, e.g., racial discrimination [29].

Based on the presented association between COVID-19-related discrimination and PTSD symptom severity in hospital staff, mediated by psychological distress, clinical and public health interventions could be strategically designed. From a mechanistic standpoint, intervening in this psychological distress may potentially be meaningful, although the proportion mediated in our study does not necessarily suggest that the portion can be actually eliminated by interventions. These interventions may include stress management strategies within the workplace context [30, 31]. In addition, performing regular mental health assessments may help identify those at risk of PTSD symptoms. On a broader scale, public health initiatives may offer anti-discrimination campaigns that help reduce the overall level of discrimination [32, 33].

Several limitations should be acknowledged here. First, our sample size was 660, which was not sufficient to obtain other estimates such as risk difference and risk ratio. While the association found in this study may be plausible, the interpretation of *β* is not as straightforward as that of risk difference and risk ratio for dichotomous outcomes. The three-item PDS has a validated cutoff [19], and analyzing the dichotomous presence of PTSD in a larger sample may provide more easily interpretable information. Second, we used self-report measures, which might have resulted in measurement bias. This applies to the exposure (COVID-19-related discrimination) and mediator (psychological distress) as well as the outcome (PTSD symptom severity). Third, we carefully controlled for potential confounders, including the preexisting outcome and mediator, using the modified disjunctive cause criterion [21]. Nevertheless, unmeasured confounders such as other psychosocial factors might still have introduced some bias. Fourth, we used the three-item PDS based on the DSM-IV, whereas the PDS-5 based on the DSM-5 is now available [34]. In future studies, incorporating such newer scales might provide more comprehensive insights, given that we indeed developed our discussion based on the DSM-5. Fifth, we handled missing data using random forest imputation. Still, a substantial proportion of the participants did not complete the follow-up survey, which may have caused bias. Sixth, we did not collect information regarding the race and ethnicity of the participants. Since discrimination might be associated with race and ethnicity, this issue might have confounded the observed associations. Finally, the hospital staff consisted of an Asian-dominant population, more than 99% of whom were vaccinated. Also, COVID-19-related discrimination might happen in a highly heterogeneous manner, dependent on regions or countries. Taken together, caution should be exercised when generalizing our findings to different populations and settings.

## Conclusions

COVID-19-related discrimination is associated with subsequent PTSD symptom severity in healthcare workers. Psychological distress may serve as an important mediator, underscoring the potential need for interventions targeting this factor.

## Data Availability

The datasets used and analyzed during the current study are available from the corresponding author on reasonable request.
